# Unilateral mydriasis: an unexpected effect of ipratropium bromide inhalation—a brief report

**DOI:** 10.1007/s00431-025-06072-1

**Published:** 2025-03-11

**Authors:** Bar Levy, Noa Ziv, Irit Krause

**Affiliations:** 1 Department of Pediatrics “C”, Schneider Children’s Medical Center of Israel, 14 Kaplan St., 4920235 Petah Tikva, Israel; 2 The Faculty of Medicine, Tel Aviv University, Tel Aviv, Israel

**Keywords:** Anisocoria, Unilateral mydriasis, Ipratropium bromide, Side effect

## Abstract

Anisocoria often raises concerns about potential underlying conditions such as intracranial hemorrhage, brain tumor, or Horner syndrome. However, iatrogenic exposures may also lead to unilateral mydriasis. A six-month-old infant was hospitalized due to acute bronchiolitis with a history of prematurity, bronchopulmonary dysplasia, and periventricular leukomalacia (PVL). He received non-invasive respiratory support, salbutamol, and ipratropium bromide by inhalation. Four days after admission, the infant exhibited an episode of sudden unilateral mydriasis with no other new signs or symptoms. Ophthalmological examination disclosed no abnormalities besides the dilated pupil, and brain CT scan showed PVL similar to the previous imaging without new pathological findings. There are several case reports of ipratropium bromide given by inhalation causing mydriasis by leakage through the inhalation mask. Ipratropium bromide is an antagonist of the muscarinic receptors. It induces bronchodilation and inhibits mucus production. One of the possible side effects of antimuscarinic drugs is mydriasis. Our patient did not exhibit any systemic signs, thus suggesting a local effect by direct contact between the eye and the offending agent. Treatment with ipratropium bromide was stopped, and within 24 h, the pupil size returned to normal. *Conclusion*: This case underscores the importance of recognizing the side effects of medications, particularly in young patients with complex medical conditions.

**Table:** 

**What is Known:**
• Unilateral mydriasis refers to unequal pupil sizes, where the abnormal pupil is dilated. • Unilateral mydriasis can result from a variety of causes, ranging from benign to serious conditions, including iatrogenic factors such as medications.
**What is New:**
• The occurrence of unilateral mydriasis induced by ipratropium bromide has been rarely reported in pediatric patients. • If unilateral mydriasis is an isolated abnormal finding and there is a history of ipratropium bromide exposure, themydriasis could be due to the ipratropium exposure.

• Unilateral mydriasis refers to unequal pupil sizes, where the abnormal pupil is dilated.

• Unilateral mydriasis can result from a variety of causes, ranging from benign to serious conditions, including iatrogenic factors such as medications.

• The occurrence of unilateral mydriasis induced by ipratropium bromide has been rarely reported in pediatric patients.

• If unilateral mydriasis is an isolated abnormal finding and there is a history of ipratropium bromide exposure, themydriasis could be due to the ipratropium exposure.

## Background

Anisocoria is defined as an inequality between the pupil sizes. The abnormal pupil could be constricted, meaning unilateral miosis, or dilated, meaning unilateral mydriasis, as seen in this case. Unilateral mydriasis typically arises from disruptions in the parasympathetic pathway. Anisocoria can occur either as a physiological variant or due to pathological factors, spanning a spectrum from benign to serious conditions[1–3].

Key causes of unilateral mydriasis include third nerve palsy (due to aneurysm, brain tumor, or intracranial hemorrhage, for example), tonic pupil, trauma, and iatrogenic factors such as medications. The causes of unilateral miosis include Horner syndrome, cluster headache, anterior uveitis, physiologic anisocoria, and pharmacologically induced miosis [1–7].

Pharmacological agents can induce either mydriasis or miosis, with mydriasis being more commonly observed. Mydriatic agents include substances or derivatives of tropane alkaloids such as atropine, scopolamine, hyoscyamine, or other antagonists of the muscarinic receptors. Medications and materials that contain such substances and thus exhibit antimuscarinic effects encompass tropicamide, cyclopentolate, glycopyrrolate deodorants, scopolamine patches, and certain herbs such as Jimson weed and Angel’s trumpet. There have been documented cases of pharmacological unilateral mydriasis induced by ipratropium bromide. However, this phenomenon has been rarely reported in pediatric patients. While systemic medications typically affect both pupils equally, a localized application can lead to anisocoria if confined to one eye [1–5].

## Case presentation

A six-month-old infant was born at 27 gestational weeks due to fetal distress secondary to hydrops fetalis. During the post-natal course, he suffered from bronchopulmonary dysplasia (BPD) and periventricular leukomalacia. His current illness presented with respiratory distress, cyanosis, and tachycardia. The patient was diagnosed with acute bronchiolitis caused by respiratory syncytial virus, identified by polymerase chain reaction testing, and a worsening of the underlying BPD.

Due to the severity of his condition, he was admitted to the pediatric intensive care unit. The treatment there included inhalation therapy with adrenaline (which was stopped before his transfer to the pediatric department), budesonide, and terbutaline sulfate, which was later switched to ipratropium bromide, due to excessive secretions from the respiratory tract. He also received systemic corticosteroids (methylprednisolone) and respiratory support via a high-flow nasal cannula.

After 6 days of intensive treatment, the patient’s condition stabilized, and he was transferred to the pediatric ward. At that point, his medication regimen included budesonide, salbutamol, and ipratropium bromide by inhalation along with methylprednisolone.

**Fig. 1:**
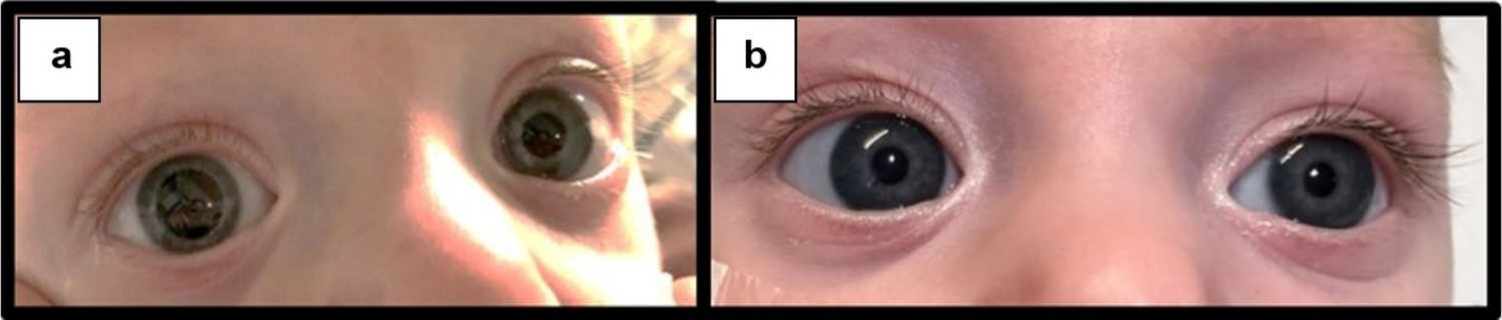
**a** The infant’s right pupil dilated and fixed, while the left pupil remained normal. **b** Pupils equal and reactive after cessation of ipratropium bromide inhalation therapy

Four days after admission to the pediatric ward, 30 min after ipratropium bromide inhalation, the infant’s right pupil suddenly became dilated and fixed, unresponsive to light, while the left pupil remained normal and responsive (see Fig. 1a). His vital signs and the rest of the neurological examination disclosed no abnormal findings. Ophthalmological examination showed no abnormalities except for the dilated pupil.

A computed tomography (CT) scan of the brain showed known prematurity-related complications, without new findings explaining the sudden pupillary abnormality.

Following a thorough review of the case, we hypothesized that the unilateral mydriasis resulted from a side effect of ipratropium bromide. The temporal relationship of ipratropium inhalational therapy, which occurred 30 min prior to pupil dilation, would suggest that the eye was exposed to a portion of the medication, likely due to an improperly fitted mask and caused unilateral pupillary dilatation.

The administration of ipratropium bromide inhalation was stopped. Within a few hours, the right pupil began to recover and react to light. After 24 h, the pupils became equal in size and reactive to light (see Fig. 1b). Notably, there were no recurrences of the event.

## Discussion

We presented here a patient with anisocoria, which refers to unequal pupil sizes. To evaluate anisocoria, the literature recommends obtaining a comprehensive medical history followed by a physical examination and a detailed neurological assessment. An ophthalmological examination is then necessary in order to determine which pupil is abnormal, to rule out traumatic damage to the iris, and to examine extraocular movements. Additionally, topical pilocarpine eye drops may be used as a miotic cholinergic test to differentiate between the potential diagnoses. If third nerve palsy is suspected, imaging studies such as a computed tomography angiogram (CTA) or magnetic resonance angiogram are recommended to detect aneurysms and other vascular abnormalities [1, 3, 8, 9].

When evaluating anisocoria, the identification of the abnormal pupil is a crucial step. This can be determined by measuring pupil sizes in both a lit room and a dark room to see if the anisocoria is more pronounced in either lighting condition. If one pupil shows a poor light reaction, meaning it does not constrict properly, and the anisocoria is greater in the presence of bright light, the abnormal pupil is the dilated one, and the problem is within the parasympathetic pathway [1, 3, 8, 9].

Unilateral mydriasis can indicate pressure on the third cranial nerve, potentially due to serious conditions such as aneurysm, brain tumor, or intracranial hemorrhage; thus, these life-threatening diseases must be excluded. However, there can also be benign causes, such as pharmacologically induced mydriasis [1–5, 8, 9].

Several pharmacological agents that can induce mydriasis include antimuscarinic medications and certain herbal substances. Localized exposure of one eye to these agents can lead to anisocoria. There have been several reports of unilateral mydriasis induced by ipratropium bromide, although such cases in pediatric patients have been rarely reported [1–5, 8, 10].

Ipratropium bromide, an antimuscarinic agent derived from atropine, works as a muscarinic receptor antagonist. As an effective bronchodilator, it is used to manage bronchospasm. The maximum effect of inhaled ipratropium bromide occurs 30–60 min after administration, with a duration of action lasting 3–6 h. Possible side effects include transient dryness of the mouth and unpleasant taste. Accidental leakage of the aerosol or droplets into the eye, often due to an ill-fitting mask, can cause unilateral mydriasis. When ipratropium bromide contacts the cornea, it exerts a parasympatholytic effect by blocking muscarinic acetylcholine receptors. This action paralyzes the sphincter pupillae and inhibits the ciliary muscles, leading to mydriasis (a fixed dilated pupil) and, if it affects only one eye, anisocoria. Systemic antimuscarinic side effects such as tachycardia and constipation are rare with typical inhaled doses because of poor systemic absorption [1, 2, 10–12].

To summarize, unilateral mydriasis resulting from the administration of ipratropium bromide or other antimuscarinic agents is a plausible and valuable diagnosis to consider. However, serious conditions must be ruled out, especially when unilateral mydriasis is not an isolated finding. In such cases, there is a need to assess the patient’s overall condition, monitor vital signs, search for additional findings (fever, decreased level of consciousness, seizures, and focal neurologic deficits), and consider brain imaging [1, 3, 8, 9].

If unilateral mydriasis is an isolated abnormal finding and there is a history of ipratropium bromide exposure, the mydriasis could be due to the ipratropium exposure. In such cases, the yield of completing a CT\CTA scan should be weighed against the possible adverse effects of radiation exposure [1, 2, 10].

If ipratropium bromide is suspected as the causative agent for mydriasis, discontinuing the medication should be the next step. If the affected pupil returns to its normal size and reacts to light, typically within 24 h, as seen in this case, then the ipratropium bromide was the likely causative agent [1].

Prevention of substance leakage from the nebulizer mask by verifying its appropriate fitting, which may be particularly challenging in younger children, is pivotal in avoiding unexpected and unwanted effects of the medications [1, 10, 12].

## Conclusion

Ipratropium bromide given by inhalation, should be considered as a causative agent in any case of isolated unilateral mydriasis in patients who have no other abnormal signs. Our report emphasizes the necessity for healthcare providers to be vigilant regarding the potential side effects of medications, particularly in very young patients with complex medical conditions.

## Data Availability

No datasets were generated or analysed during the current study.
